# Restoration of degraded grasslands, but not invasion by *Prosopis juliflora,* avoids trade-offs between climate change mitigation and other ecosystem services

**DOI:** 10.1038/s41598-020-77126-7

**Published:** 2020-11-24

**Authors:** Purity Rima Mbaabu, Daniel Olago, Maina Gichaba, Sandra Eckert, René Eschen, Silas Oriaso, Simon Kosgei Choge, Theo Edmund Werner Linders, Urs Schaffner

**Affiliations:** 1 Kenya Forestry Research Institute (KEFRI), Baringo Sub-Centre, P.O. Box 57-30403, Marigat, Kenya; 2 Institute for Climate Change and Adaptation (ICCA), University of Nairobi, P.O. Box 30197-00100, GPO, Nairobi, Kenya; 3 Faculty of Humanities and Social Sciences, Chuka University, P.O. Box 109-60400, Chuka, Kenya; 4 Centre for Development and Environment (CDE), University of Bern, Mittelstrasse 43, Bern, Switzerland; 5 CABI, Rue des Grillons 1, Delémont, Switzerland; 6 Institute of Plant Sciences, University of Bern, Altenbergrain 21, Bern, Switzerland; 7 Senckenberg Biodiversity and Climate Research Centre (SBiK-F), Senckenberganlage 25, 60325 Frankfurt am Main, Germany

**Keywords:** Ecology, Biodiversity, Ecosystem services, Grassland ecology, Invasive species, Climate-change mitigation, Climate sciences, Carbon cycle

## Abstract

Grassland degradation and the concomitant loss of soil organic carbon is widespread in tropical arid and semi-arid regions of the world. Afforestation of degraded grassland, sometimes by using invasive alien trees, has been put forward as a legitimate climate change mitigation strategy. However, even in cases where tree encroachment of degraded grasslands leads to increased soil organic carbon, it may come at a high cost since the restoration of grassland-characteristic biodiversity and ecosystem services will be blocked. We assessed how invasion by *Prosopis juliflora* and restoration of degraded grasslands in a semi-arid region in Baringo, Kenya affected soil organic carbon, biodiversity and fodder availability. Thirty years of grassland restoration replenished soil organic carbon to 1 m depth at a rate of 1.4% per year and restored herbaceous biomass to levels of pristine grasslands, while plant biodiversity remained low. Invasion of degraded grasslands by *P. juliflora* increased soil organic carbon primarily in the upper 30 cm and suppressed herbaceous vegetation. We argue that, in contrast to encroachment by invasive alien trees, restoration of grasslands in tropical semi-arid regions can both serve as a measure for climate change mitigation and help restore key ecosystem services important for pastoralists and agro-pastoralist communities.

## Introduction

Soils are the largest terrestrial carbon reservoir containing more carbon than vegetation and the atmosphere combined^1^. Moreover, they present a relatively stable carbon stock, as compared to the more transitory carbon stock in woody biomass^2^. Yet, soil organic carbon (SOC), which makes up about two thirds of global soil carbon^3^, is sensitive to land degradation^4^, with significant negative consequences for soil quality and productivity and an exacerbation of greenhouse gas emissions^5^. Since halting land degradation and restoring degraded soils and their associated services is essential for building agro-ecological systems that meet global development goals^6,7^, the management of soil resources will have wide-ranging consequences on human well-being for generations to come^8^.

Grasslands, which comprise approximately 40% of Earth’s natural vegetation^9^, contain a substantial amount of the world’s SOC^10^. In addition, they provide habitat for a substantial diversity of animals and plants and support other ecosystem services (ES), including the regulation and storage of water flows, forage for livestock production, and tourism^11^, thereby contributing substantially to the livelihoods of over one billion of people worldwide^12,13^. Yet, grasslands are under severe threat from degradation and conversion to other land uses^14^, which limits their potential to provide these essential services and functions. Degradation of grass-dominated ecosystems and the concomitant loss of SOC and other ES is particularly prevalent in arid and semi-arid regions in Sub-Saharan Africa (SSA) due to a set of interlinked factors, including a growing population, overgrazing, invasion by alien plant species^15^ and a lack of appropriate policies^16^ among others.

In response to the program for Reducing Emissions from Deforestation and Forest Degradation (REDD +) and the Kyoto Protocol's Clean Development Mechanism (CDM), afforestation of degraded grasslands has been put forward as a legitimate climate mitigation strategy^17,18^. Some countries that have commercialized carbon credits under the Kyoto Protocol even consider promoting the cultivation of invasive alien tree species^19,20^. However, the extent to which afforestation or tree invasions lead to increased soil C stocks depends on prior land use or land cover, climate and the tree species, thus assessments relying on carbon stored from woody plant invasions to balance emissions may be incorrect^21^. In fact, the expansion of trees or shrubs into grasslands can also lead to a depletion of valuable soil carbon stocks^21–23^. The emphasis on aboveground C stocks may also be due to the fact that most studies on soil carbon restricted their analyses to surface soils (usually upper 15–30 cm), thereby ignoring the potential of grassland soils to store SOC at greater depth^24^. When deciding on soil carbon management actions, the co-benefits and trade-offs with other ES should be identified and considered to promote ecosystem service multifunctionality^25^. Even in cases where encroachment by trees in tropical savannas leads to increased C stocks, it may come at a high cost to biodiversity and other ES^26,27^. Considering the potential of healthy grassland soils to store large amounts of C, an alternative climate mitigation strategy would be to restore degraded grasslands and implement sustainable grazing management^28^. Because grasslands have greater carbon allocation to root systems than forests, restoration of the former may potentially replenish depleted SOC stocks on degraded land as quickly as encroachment by woody species^29^.

Here we assessed the impact of grassland degradation and 25–35 years of either encroachment of the invasive alien tree *Prosopis juliflora* (Sw.) DC (hereafter referred to as *Prosopis*) or grassland restoration on SOC, biodiversity and herbaceous biomass in Baringo County, a tropical semi-arid region in Kenya. *Prosopis*, which is native to Central America, was introduced into various countries in Eastern Africa in the 1960s and 1970s in an attempt to combat land degradation and to provide additional services, such as firewood and fodder for livestock^30^. Soon after its introduction, *Prosopis* started to spread out from the plantations and to invade surrounding habitats, including grasslands^31^. We quantified soil organic carbon stocks (down to 1 m belowground), plant species richness and herbaceous biomass in pristine grassland, degraded grassland, grassland moderately and heavily invaded by *Prosopis*, and in restored grassland in Baringo County. We hypothesized that i) SOC decreases with increasing soil depth but that this pattern varies among land cover types, ii) restoration of grasslands is as effective in carbon sequestration as *Prosopis* forest, and iii) grassland restoration, in contrast to *Prosopis* encroachment, also promotes fodder for livestock production and biodiversity, thus avoiding carbon-fodder or carbon-biodiversity trade-offs.

## Results

### Soil depth and land cover type effects on SOC concentration and SOC per volume

**Figure 1:**
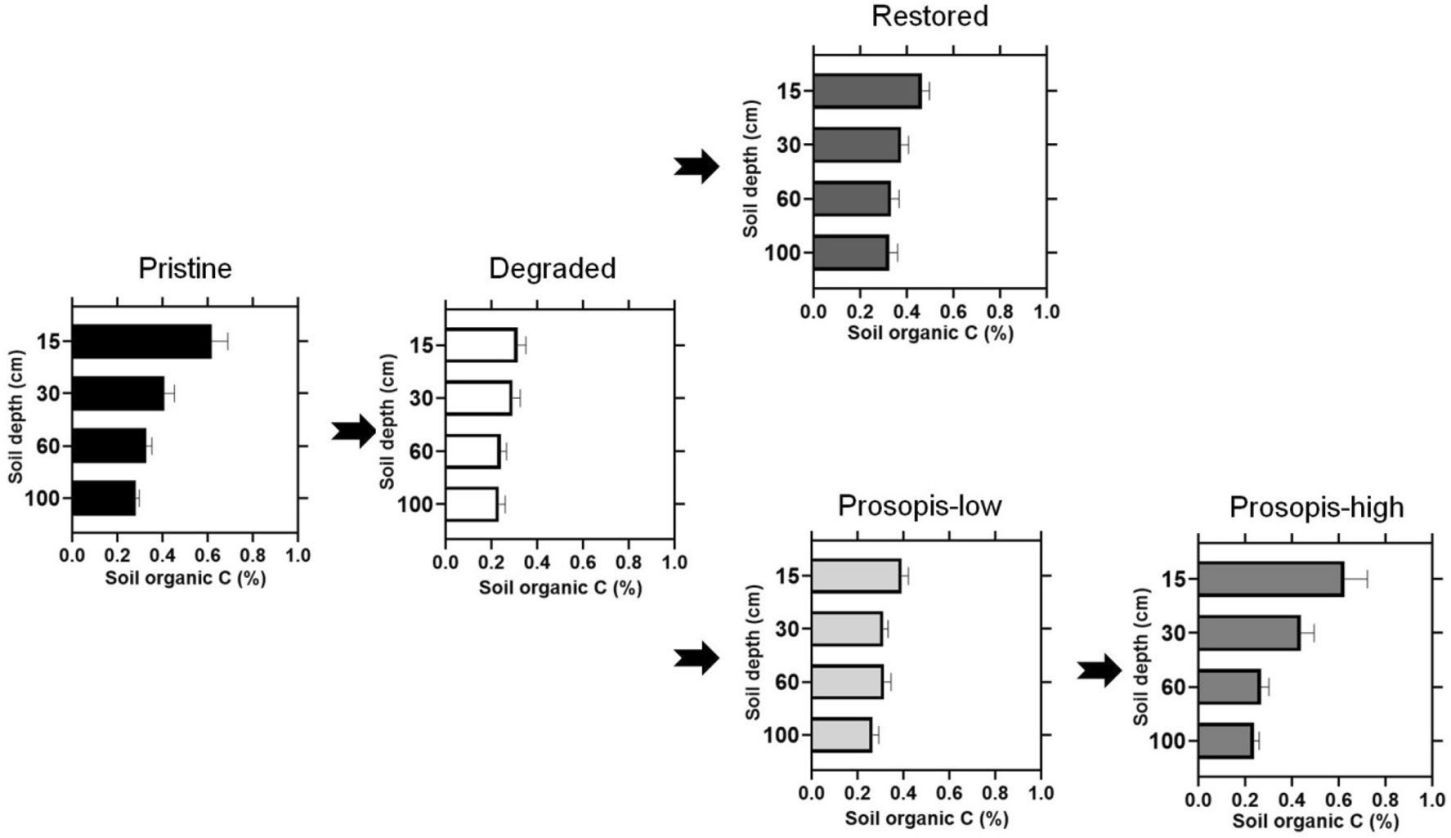
Soil organic carbon concentration (%SOC) for the five land cover types and four soil depth increments. Error bars indicate standard errors. The arrows represent a hypothetical transition from one land cover state to the next over time.

Both SOC concentration (%SOC) and SOC per volume (g C cm^-3^) were significantly affected by soil depth (%SOC: F_3, 174_ = 36.63, p < 0.001; SOC per volume: F_3, 174_ = 37.80, p < 0.0001) and land cover type (%SOC: F_4, 58_ = 4.82, p = 0.002; SOC per volume: F_4, 58_ = 6.15, p = 0.003), Fig. 1 and Supplementary Figure S1 online. Moreover, differences in %SOC and SOC per volume across land cover types depended on soil depth (interaction effect %SOC: F_12, 174_ = 2.26, p = 0.011; interaction effect SOC per volume: F_12, 174_ = 1.98, p = 0.029; Supplementary Tables S1, S2 online). Land cover type strongly influenced %SOC and SOC per volume in surface soils (0–30 cm: F_4, 58_ = 7.72, p < 0.001) and to a lesser extent also in deeper soil layers (31–100 cm: F_4, 58_ = 3.15, p = 0.021). Highest values for %SOC and SOC per volume in the top layer were found in pristine grassland and *Prosopis*-high land cover types, while at higher depth the highest values were found in restored and pristine grassland (Supplementary Tables S1, S2 online). Both %SOC and SOC per volume declined with increasing soil depth down to 1 m (Supplementary Figures S2, S3 online**)**. Greatest reductions in %SOC and SOC per volume across depth were observed in *Prosopis*-high (62% each) and pristine grasslands (54% and 49%, respectively), while the smallest reductions occurred in degraded (26% and 30%, respectively) and restored grasslands (30% and 33%, respectively). Overall, the largest variation in %SOC and SOC per volume across land cover type was found in the surface soils (0–15 cm).

Soil bulk density was strongly affected by land cover type (F_4, 58_ = 5.11, p = 0.001). It was highest in pristine grasslands, intermediate in restored and degraded grasslands and lowest in *Prosopis* invaded plots (Table 1). The effect of soil depth on bulk density was not consistent (F_3, 174_ = 1.55, p = 0.204), but varied among land cover type (interaction effect: F_12, 174_ = 2.48, p = 0.005; Supplementary Table S3 online). While bulk density in pristine grasslands increased with increasing soil depth, it decreased or showed no consistent trend in the other land cover types (Supplementary Table S3 online).

**Table 1:** Sampling sites characteristics and mean (Standard Error) of the various variables per land cover type. Land cover types sharing a letter are not significantly different at **α** = 0.05. Age was estimated using a combination of information sources and criteria such as literature review^95–97^, time series maps for the study area^31^, and consultations with key informants in the study area. Vegetation cover was determined using *Prosopis* fractional cover map for *Prosopis* plots, as well as land use/land cover maps^31^ and field observations for the other land cover types.

Land cover	Age (years)	Vegetation cover (%)	n	% SOC 0–100 cm	Bulk density (g cm^-3^)	SOC per volume (g cm^-3^)	Total SOC t ha^-1^ 0–100 cm	Species richness /225 m^2^	Herbaceous biomass (g m^-2^)
Pristine	> 70	> 80	10	0.091 (0.005)	1.37 (0.02)	0.0053 (0.0006)	49.76 (2.28) *c*	18.30 (0.58) *c*	1281.7 (213.01) *b*
Degraded	> 70	< 5	16	0.064 (0.005)	1.26 (0.03)	0.0031 (0.0003)	31.52 (3.04) *a*	8.62 (1.19) *a*	147.3 (42.92) *a*
*Prosopis*-low	10—15	< 30	12	0.077 (0.005)	1.21 (0.02)	0.0037 (0.0004)	36.99 (2.51) *ab*	12.67 (1.03) *bc*	59.9 (16.88) *a*
*Prosopis*-high	25—35	> 80	10	0.083 (0.006)	1.20 (0.03)	0.0043 (0.0005)	40.05 (1.28) *abc*	9.40 (0.95) *ab*	5.7 (1.20) *a*
Restored	25—35	> 80	15	0.089 (0.006)	1.26 (0.02)	0.0044 (0.0004)	44.68 (3.77) *bc*	9.40 (1.37) *ab*	678.0 (85.36) *b*

### Total soil organic carbon

**Figure 2:**
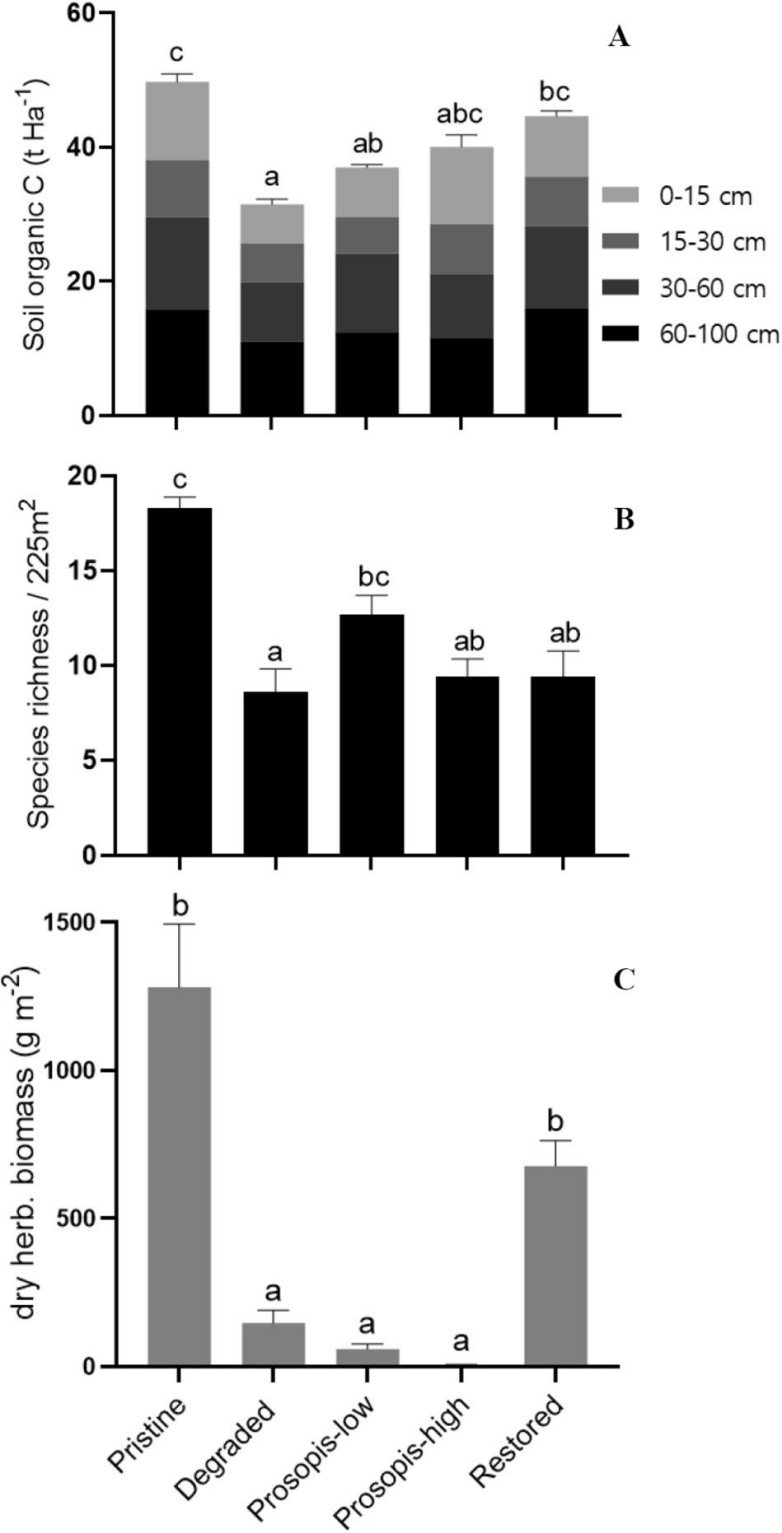
Total soil organic carbon in tonnes per hectare at four soil depth increments from surface to 1 m below ground (**A**), species richness per plot (225 m^2^) (**B**) and dry weight of herbaceous vegetation g m^-2^ (**C**), shown for the five land cover types. The error bars indicate standard errors. Land cover types sharing a letter are not significantly different at **α** = 0.05.

When adjusting %SOC to the different depths of soil increments, %SOC figures to one meter depth were highest in pristine grasslands, followed by restored grasslands, and lowest in degraded grasslands (Table 1). Similarly, total SOC to 1 m depth was strongly influenced by land cover type (*F*_*4, 58*_ = *5.53, p* < *0.001*), with pristine grasslands having the highest total SOC stock (49.76 ± 2.28 t C ha^-1^), followed by restored grasslands (44.68 ± 3.77 t C ha^-1^), high *Prosopis* densities (40.05 ± 1.28 t C ha^-1^), low *Prosopis* densities (36.99 ± 2.51 t C ha^-1^) and degraded grasslands (31.52 ± 3.04 t C ha^-1^; Fig. 2A). Total SOC in degraded grassland was significantly lower than that in pristine (-37%) and restored grasslands (-29%), while total SOC in both low and high *Prosopis* densities did not differ from any of the other land cover types (Supplementary Table S4 online).

### Plant species richness and land cover-specific indicator species

In the 63 sampled plots comprising the five land cover types, a total of 81 different plant species were recorded. Plant species richness per plot varied significantly among land cover types (*F*_*4, 58*_ = *8.66, p* < *0.001*). It was approximately twice as high in pristine than in degraded grasslands (Table 1; Fig. 2B). Degraded grassland had lower plant species richness than pristine grasslands and low density *Prosopis* areas, but did not differ from high density *Prosopis* and restored areas. Plant species richness in low density *Prosopis* areas did not differ from that in pristine grasslands (Supplementary Table S5 online).

We identified four indicator species that were characteristic of three land cover types in the study area: *Portulaca oleracea* (degraded grassland), *Cynodon dactylon* and *Waltheria indica* (pristine grasslands), and *Cenchrus ciliaris* (restored grasslands) (Table 2). We did not find any plant species associated with *Prosopis* invaded areas.

**Table 2:** Indicator species for three land cover types, indicator value and number of plots containing species in the five land cover types. Significance level (**α** = 0.05).

Indicator species	Lifeform	Land cover	Indicator value	*p-value*	Number of plots containing species				
					Pristine	Degraded	*Prosopis*-low	*Prosopis*-high	Restored
*Portulaca oleracea*	Annual	Degraded	0.08	0.02	0	12	2	0	2
*Cynodon dactylon*	Perennial	Pristine	0.05	0.05	10	0	7	0	2
*Waltheria indica*	Perennial	Pristine	0.05	0.04	10	0	0	0	1
*Cenchrus ciliaris*	Perennial	Restored	0.09	0.03	3	7	3	0	14

### Herbaceous biomass

Dry herbaceous biomass varied significantly among land cover types (*F*_*4, 58*_ = *33.97, p* < *0.001*). Herbaceous biomass was almost six times higher in pristine grassland than in degraded or lightly invaded areas, and more than 200 times higher than in highly invaded areas (Fig. 2C). Herbaceous biomass in pristine and restored grasslands did not significantly differ from each other (Supplementary Table S6 online).

## Discussion

Our results provide evidence that restoration of degraded grasslands in the semi-arid regions of Baringo County, Kenya, is at least as effective in replenishing SOC pools as encroachment by the invasive *Prosopis* tree. Thirty years of grassland restoration increased SOC pool almost to the level of pristine grasslands. In addition, and in contrast to *Prosopis* encroachment, grassland restoration also promotes fodder for livestock production, thus avoiding carbon-fodder trade-offs. Recovery of plant species richness requires more time and/or targeted grassland management interventions which promote restoration of the characteristic biodiversity.

### Soil organic carbon stocks at depth

As found in other studies assessing SOC at different soil depths^29,32,33^, both %SOC and SOC per volume decreased with increasing soil depth across all land cover types. The greatest decline in SOC per volume occurred from 0–15 to the 15–30 cm depths, similar to observation made on the Hawaiian Islands^33^. Nevertheless, in terms of total SOC per ha, our results corroborate other studies in grasslands that show that deeper soils can store substantial amounts of SOC^24,29^. For example, of the total SOC found in pristine grassland soils, some 59.6% were found between 30 and 100 cm depth. These values closely correspond with Lal et al.^34^ who estimated that globally, ⁓55% of the SOC to 1 m depth lies below 30 cm depth. Using global data sets of soil profiles, Jobbágy et al.^29^ estimated that in grasslands the amount of SOC in the second and third meters was some 43% of that in the first meter. The substantial storage of SOC in deeper soils across all land cover types considered in this study underpins the importance of sampling beyond the threshold of 30 cm belowground^24,35^.

### Soil organic carbon under different land cover types

Our estimated total SOC values for pristine and restored grasslands to 1 m depth (49.76 and 44.68 t C ha^-1^, respectively) are slightly lower than those reported by Adams et al.^36^ for savanna and thorn scrub and scrub woodland biomes (54 and 60 t C ha^-1^, respectively), but within the FAO-UNESCO soil unit range of 4.2 – 6.2 kg C m^-2^ estimated for Xerosols^3^. The carbon values of *Prosopis* invaded areas (both in low and high cover areas) were within the range of estimated SOC values for low and dense *Prosopis* cover in the native range in Texas, USA^37^.

While long-term variation in organic carbon accumulation in soils largely result from factors affecting climate, geology and soil formation^33,36^, changes in land use / land cover are at play over shorter periods^3,34^. For example, plant functional groups with different allocation of photosynthates to above- and belowground plant parts and with different root architecture are known to affect the total amount and the vertical distribution of SOC profiles^3,29^. Our findings provide evidence that degradation of natural vegetation in the semi-arid parts of Baringo County and re-establishment of vegetation have led to major changes in total SOC stocks and SOC profiles to 1 m depth. First, total SOC stock in degraded grasslands was 37% lower than that in pristine grasslands. This value is considerably higher than that reported in a worldwide review by Dlamini et al.^4^ for degraded grasslands in arid and semi-arid regions (-16%), but studies in South Africa revealed losses of SOC stocks which were comparable or even higher than those reported in our study. For example, Dlamini et al.^4^ found a 79% SOC loss in grasslands in KwaZulu-Natal Province with < 5% vegetation cover and a 42% SOC loss in grasslands with < 50% cover, while Baer et al.^38^ reported a 56% loss in total SOC due to cultivation of grasslands in the South African Highveld. Based on field data collected in Serengeti National Park, Tanzania, Ritchie^39^ modelled the effect of grazing intensity on SOC and predicted that SOC increases at intermediate grazing intensity but then declines rapidly at the highest grazing intensities. We attribute our lower SOC levels in degraded grasslands largely to the grazing pressure by livestock, because in our study area, grazing pressure is above the threshold where SOC is expected to decline rapidly (see below).

The other significant difference among land cover types in total SOC stocks was between degraded and restored grasslands. Notably, 30 years of grassland restoration led to an increase in SOC in all soil depth increments, including the depth increment at 60–100 cm. At the COP21, the ‘4 per 1000 – Soils for Food Security and Climate’ initiative was launched to promote mitigation of climate change through the annual increase in soil organic carbon by 0.4% in the top 30–40 cm of agricultural, grasslands, pastures and forest soils^40,41^. The difference between degraded and 30 years old restored grasslands in our study area correspond to an average annual increase in total SOC of 1.4% ([SOC in restored grasslands – SOC in degraded grasslands]/years since restoration started) in both the top 30 cm as well as in the top meter, which is significantly above the goal of the ‘4 per 1000′ initiative. Our higher values of annual increase are in line with the findings by Corbeels et al.^42^, that SOC storage rates under conservation agriculture and multistrata agroforestry systems are actually higher than the set goal of 0.4%. Increases in SOC due to restoration of degraded grasslands in semi-arid and arid regions of Africa have also been reported by Chaplot et al.^43^ (33% increase within two years of livestock exclosure and NPK fertilization in KwaZulu-Natal Province of South Africa) and by Oduor et al.^44^ (27% increase in 3 to > 20 years old grassland exclosures in West Pokot County, Kenya). It is notable that seventeen of the twenty countries in the world with more than 70% of grassland area are found in Sub-Saharan Africa^11^ and nearly a quarter of sub-Saharan Africa contains land classified as ‘severely degraded’^45^. Thus, while grasslands in semi-arid and arid regions may have a lower potential to sequester SOC than wetlands when expressed on a per-unit-area basis^41^, sustainable grassland management is likely to play a key role for SOC storage in Sub-Saharan Africa due to the very large area of this ecosystem and thus for climate change mitigation. For example, assuming an accumulation rate of SOC of 1.4% (as estimated for the restored grasslands in this study), the restoration of the currently over 8,500 ha of severely degraded land in the Njemps Flats, Baringo County, would translate into an annual sequestration of C of approx. 3,700 tons C over the next 30 years.

The *Prosopis*-low and the *Prosopis*-high cover areas had intermediate levels of total SOC stocks which neither differed significantly from those of pristine and restored grasslands nor from those of degraded grasslands. The fact that SOC stocks in pristine and in restored grasslands tended to be even higher than those in *Prosopis* invaded sites may at least partly be attributed to the fact that grassland soils have organic matter levels at least twice as high as forests because grassland biomes add organic matter to topsoil from both roots and above-ground resulting from annual die back^46^. The grasses *Cynodon dactylon* and *Cenchrus ciliaris*, which were indicative of pristine and restored grasslands, respectively, are known for having deep root systems extending up to 2 m into the soil profile^47,48^. In particular, *C. ciliaris*, one of the grasses sown in the Rehabilitation of Arid Environments Charitable Trust (RAE Trust) grassland restoration project, is reported to build 60–100 cm long roots within 4–16 months after seeding^49^.

Studies in the native and in the invaded range revealed that the effect of encroachment by woody species like *Prosopis* on SOC is context-dependent. Jackson et al.^21^ and Mureva et al.^50^ found a negative relationship between precipitation and changes in SOC content when grasslands were invaded by woody vegetation, with drier sites gaining and wetter sites losing SOC. Due to the fact that overall losses of SOC at the wetter sites were substantial enough to offset increases in plant biomass carbon, Jackson et al.^21^ suggested that assessments relying on carbon stored from woody plant invasions to balance emissions may be incorrect. Our study provides evidence that the effect of *Prosopis* encroachment on SOC is also context-dependent. If *Prosopis* invades already degraded ecosystems, then it tends to increase SOC, particularly in the top 30 cm. In contrast, if *Prosopis* invades pristine or restored grasslands, it is likely to have no or potentially even a negative effect on SOC. Encroachment of grasslands by *Prosopis* leads to increased C stored in above-ground plant biomass, but plant biomass C in *Prosopis* invaded ecosystems is lower than the SOC pool in the upper 30 cm of the soil and considerably lower than the SOC pool down to 1 m (Fig. 2; Supplementary Table S7 online)^51^. Moreover, plant biomass C stocks are more vulnerable to loss from fire, biomass harvesting and other disturbances^52,53^. It is noteworthy that the sum of SOC and above-ground C of *Prosopis*-high cover plots does not exceed that of pristine grasslands.

In contrast to pristine and restored grasslands, *Prosopis* encroachment, which occurred in Baringo often in already degraded land, primarily accumulated SOC in the top 30 cm, which represents 65% and 55% of the total SOC in *Prosopis*-high cover and *Prosopis*-low cover plots, respectively. This effect can be explained by the fact that *Prosopis* exhibits two rooting systems – the main taproot and a dense network of extensive lateral roots^54^. In mature *Prosopis* trees, the main taproot is associated with a high number of smaller roots at a depth of approximately 1 m^54^, while the lateral roots are concentrated in the upper 30 cm of the soil profile^55^.

### Grassland management to create synergies between carbon sequestration, biodiversity and ecosystem services

The multiple values of grassland ecosystems to humanity have long been recognised, ranging from direct benefits of agricultural production to indirect ES such as the regulation of climate and water quality, the provision of plants for medicinal purposes and pollination services^11^, among others. As such, grasslands are arguably one of the most valuable biomes for ecosystem service provision, but they are also among the most threatened by anthropogenic activities^56^. Many of the world’s grasslands are being lost due to land use change or degraded by poor grazing management or invasive species, thereby undermining their capacity to support biodiversity and provide ES^18^.

In Baringo County, human population growth, land use changes and unsustainable grazing management, combined with communal land tenure system and a lack of enforcement of land use rights, have led to large-scale degradation of grasslands and other ecosystems, with serious consequences for biodiversity and the provisioning of multiple ES^57–59^*. Prosopis* was planted at multiple sites in Baringo to mitigate the challenges of desertification, including sand storms, and to provide services such as wood and fodder for livestock^60^. However, *Prosopis* started to escape from the plantations and to invade degraded land as well as cropland, semi-natural ecosystems and protected areas in the surroundings, and this invasion process is ongoing in Baringo^31^ as well as in other parts of Sub-Saharan Africa^61^. In both Baringo, Kenya and Afar Region, Ethiopia, over 30% of the grasslands present in the mid-1980s have disappeared and are now covered by *Prosopis*^31,62^. Prosopis is a so-called transformer species^63^ i.e. it can also invade undisturbed habitats^64^. However, the rapid invasion in Eastern Africa is likely to be further promoted by land degradation as well as by regular and seasonal migration of livestock and severe flooding events, which both contribute to seed dispersal^31^. Climate change effects such as increasing frequencies of drought events may further accelerate Prosopis invasion since their deep-reaching roots allow them to tap groundwater in areas where most native species cannot^65^. Also, rising atmospheric carbon dioxide levels have been shown to promote the growth of woody C3 plants over C4 grasses in the African savannas^66,67^, but their impact on Prosopis invasion remains to be elucidated.

The challenge for sustainable land management in Baringo and other parts of the semi-arid and arid land in Sub-Saharan Africa is to restore ecosystem multifunctionality^25^ to cover the needs of the stakeholders benefitting from healthy ecosystems. However, there are potential trade-offs between certain ES and biodiversity, or among ES. Carbon mitigation programs that promote forest cover in tropical grasslands and savannas cannot be assumed to provide net benefits for conservation or the provision of ecosystem multifunctionality^19,20^. For example, Abreu et al.^26^ showed that fire suppression in savannas of the Brazilian Cerrado increased carbon stocks but was associated with acute biodiversity loss. Similarly, encroachment of degraded tropical grasslands in Baringo by *Prosopis* increases carbon stocks and the availability of wood, but it threatens biodiversity across multiple trophic levels^64^, increases mosquito densities^68^, reduces the provisioning of herbaceous fodder for grazers^69^ and water^65,70^ and negatively affects tourism^71^ and limits access to water points, pasture, croplands and fishing grounds^72^. In arid and semi-arid regions, the high water consumption by Prosopis is of particular concern, as it decreases the groundwater recharge and thus seriously affects the water available to households in invaded ecosytems^65^. While *Prosopis* trees may provide pods for livestock feed, they should only be used as a fodder supplement and thus cannot replace the loss of native forage plants^73^.

Our results provide evidence that the replenishment of the SOC stocks through restoration of degraded grasslands can be achieved within 20–30 years and does not lead to multiple trade-offs with biodiversity or ES. Restoration of grasslands also increased fodder almost to the level of pristine grasslands, a key ecosystem service for the many pastoralist and agro-pastoralist communities inhabiting grasslands in Sub-Saharan Africa. In line with our findings, Mureithi et al.^74^, showed that the restored grasslands in Baringo, which were managed as communal enclosures, provide a source of income through the sale of fattened livestock, harvested grass seeds, hay, honey and charcoal, and that total enclosure income increased with time. The extent to which grassland restoration will increase primary productivity and SOC will depend on socio-economic factors, including land tenure systems and enforcement of land use rights, affecting the level and type of grazing management. A report by Byrnes et al.^28^ showed that grasslands with rotational grazing had SOC stocks higher than those of grasslands with continuous grazing and comparable with those of grasslands with no grazing. Moreover, and in contrast to encroachment by *Prosopis* or other woody invasive species, restoration of grasslands does not reduce availability or accessibility of surface or ground water^65,70^ and thus does not exacerbate effects of climate change on semi-arid or arid ecosystems.

In Baringo, 30 years of grassland restoration was not sufficient to restore plant species richness. This may be partly due to the long history of land degradation in the study area^75^ and a likely depletion of the soil seed bank. In Afar Region, Ethiopia, experimental studies to restore grasslands after removal of *Prosopis* led to the establishment of species-rich communities within a few years (B. Megersa, unpubl. results). Restoration of high plant diversity may considerably increase carbon capture and storage rates on degraded and abandoned land^76^. Thus, in order to accelerate the restoration of species-rich plant communities, particularly on land with a long degradation history, reseeding of a diverse set of native species should be considered. Areas with low *Prosopis* invasion tended to have a higher species richness than degraded, restored and areas with high *Prosopis* invasion. At low densities, *Prosopis* trees may provide shade for annual plants to grow underneath their canopy. Annual plants do not, however, provide the same ES as perennial plants, e.g. the bunch grass *Cenchrus ciliaris*, as they do not build up extensive below-ground biomass and the above-ground biomass dries up soon after the rains stop. Furthermore, *Prosopis* is highly prolific hence cover densities transition rapidly which suppresses the understory vegetation. Plant species richness in *Prosopis*-invaded habitats drops to very low levels once *Prosopis* has reached 50% cover^64^, which has been attributed to competitive exclusion of the slow-growing shade-intolerant herbaceous savanna species by *Prosopis*, either due to its allelopathic effects or through competition for water during the dry seasons^77^.

## Conclusions

The importance of managing grasslands to optimise carbon sequestration for climate change mitigation is widely recognised^24^. Our findings provide evidence that grassland degradation depletes SOC stocks to 1 m depth, but that restoration of degraded grasslands has the potential to replenish SOC stocks at a rate higher than targeted by international initiatives such as the ‘4 per 1000′ initiative. Moreover, refilling of the SOC stocks through restoration of grasslands does not come at the expense of the productivity at the herbaceous layer and numerous other ecosystem services necessary to meet the demands of local (agro-)pastoralists and other stakeholders. Encroachment of degraded semi-arid grasslands by *Prosopis* increased the availability of wood and can also refill C stocks, but primarily in the upper 30 cm and at the expense of fodder for livestock and numerous other ecosystem services, including water availability^65,70^. We therefore propose that efforts to reverse land degradation in Baringo and other parts of Sub-Saharan Africa should consider restoration of historical grasslands and their associated ecosystem services and their sustainable embedding in a mosaic of other ecosystems, e.g. shrubland and forests consisting of non-invasive woody species^78^***.***

## Methods

### Study area

**Figure 3:**
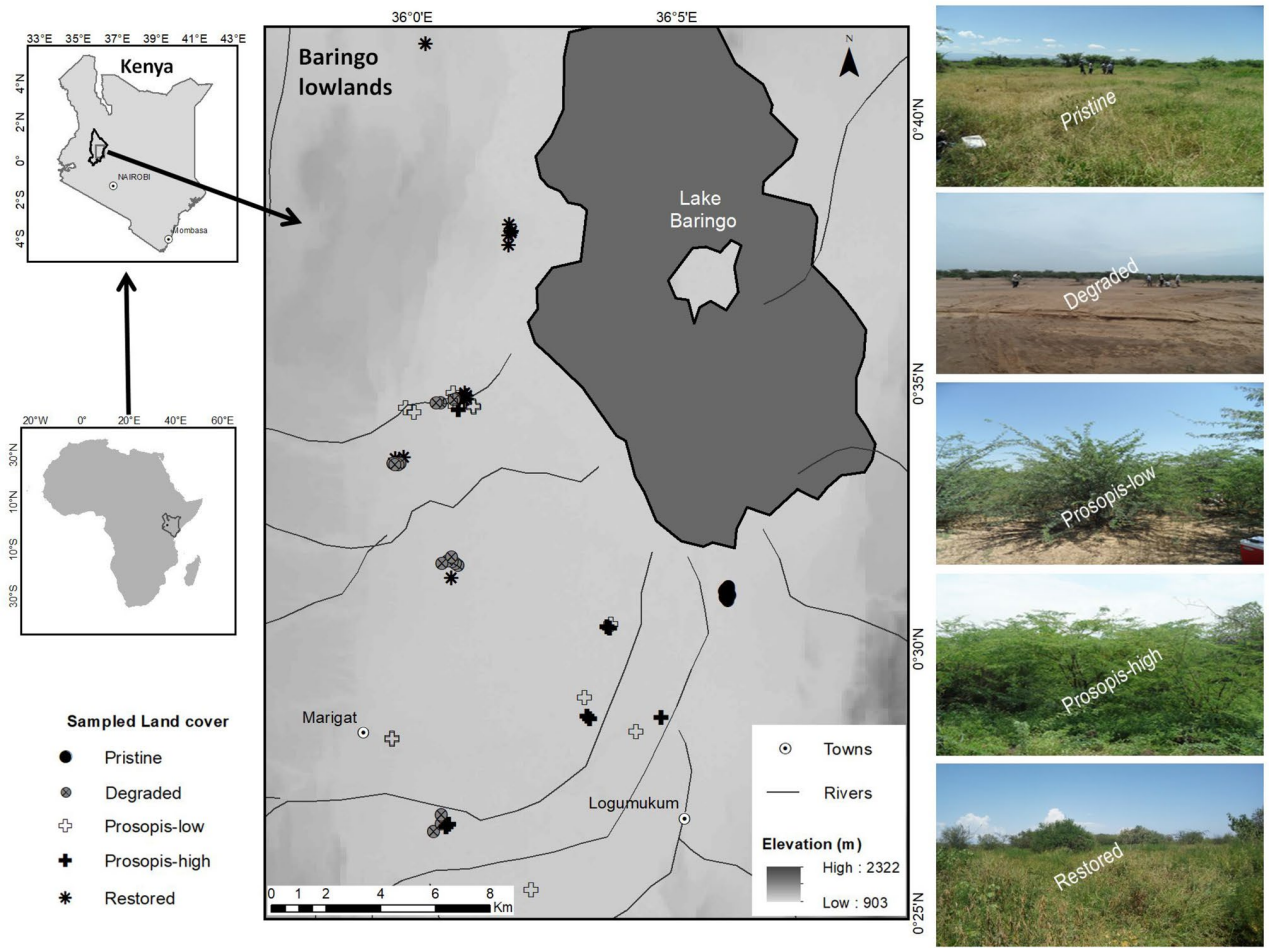
Overview of Baringo lowlands (Njemps Flats) in Kenya, the location of the sampled plots and sample photos for each land cover type. The large map (middle), is displayed on a digital elevation model generated by the Shuttle Radar Topography Mission (SRTM), provided by United States Geological Survey (USGS) available at https://earthexplorer.usgs.gov/. The two inset maps on the left for Kenya and Africa were generated using GIS data downloaded from World Resources Institute (www.wri.org) and ESRI (www.arcgis.com) respectively. The map was designed using ArcMap version 10.2.2.

The study was conducted in the semi-arid lowlands (Njemps Flats) of Baringo County, located along the Great Rift Valley system in Kenya (Fig. 3). The study area extends from latitude 0° 10′ N to 0° 50′ N and longitude 35° 20′ E to 36° 20′ E, covering an area of approximately 180,000 ha. The Njemps Flats are slightly undulating, with an average altitude of 700 m a.s.l. They are surrounded by Laikipia Escarpment on the east and Tugen Hills and Elgeyo Escarpment on the west, ridges and plateaus of the Lake Baringo catchment with peaks of over 3000 ma.s.l^79^. The average annual temperature and precipitation are 24.6 °C and 671 mm, respectively^80^. Presently, the vegetation is predominantly a woody mixture of indigenous and exotic species. It ranges from *Vachellia*-dominated deciduous shrubland on the valley floor to the evergreen forests in the highlands^52^. *Prosopis* currently dominates the lowland flats, the shores of Lake Baringo and further southwards to Lake Bogoria`s western shoreline^31,79^. Historically, the lowland flats consisted of a mosaic of grasslands and *Vachellia*-dominated savanna^81^. The study area is essentially a rangeland with few isolated pockets of dryland subsistence agriculture and small-scale irrigation in Marigat^79^. The major social-economic activities are livestock production, and bee-keeping, and more recently charcoal production^82^. A more detailed description of the study area is provided by Mbaabu et al.^31^.

During the last 300 years, Baringo has experienced severe land degradation, particularly in the lowland areas^57–59,83^. This problem was further exacerbated by rapid accumulation of livestock by resident households from 1900 onwards^57^. Over the centuries, the situation remained unchanged, eventually culminating in acute scarcity of basic ecosystem goods and services^15^. This prompted the introduction of *Prosopis* species in these lowland areas between 1982 and 1983 through the Fuelwood Afforestation Extension Project^60^ to mitigate firewood scarcity and desertification^60^. Due to its invasive nature, *Prosopis* rapidly spread from the initial plantations to the surrounding areas and currently covers over 18,000 ha of land, of which more than 5000 ha used to be barren land at the time *Prosopis* was introduced in Baringo County^31^. This tree has also encroached some previously productive land such as grasslands, croplands and other native vegetation with adverse implications for biodiversity and rural livelihoods^31^. For instance, the current estimate of the remaining patches of pristine grassland is about 21 ha, while it covered approximately 7000 ha in the mid-1980s^31^.

In parallel, grassland restoration programmes by reseeding degraded land with native grass species were initiated in the early 1980s^84,85^. The RAE Trust works with the local pastoralist community to reclaim degraded semi-arid land for more sustainable livelihoods^86^.Currently, over 4,850 ha of degraded land in Baringo lowlands have been put under restoration, of which more than 1,600 ha^87^ are located within the study area.

Baringo lowland is characterised by complex soils with diverse textures and drainage conditions which have developed into alluvial deposits^79^. Its geological products are a result of past tectonic events of faulting, warping and volcanic eruptions associated with the formation of the Rift Valley. The major soil types present are clay, loam, silt and sand^79^. Soil sampling across all land cover types was done in relatively loamy soils as this category generally supports most vegetative growth.

### Experimental design and selection criteria for sampling plots

Five land use/cover types representing common earth cover and distinct land use activities within the study area were identified, namely pristine grasslands, degraded grasslands, *Prosopis*-low (low cover), *Prosopis*-high (dense cover) and restored grasslands (reference photos in Fig. 3). The choice of these land cover types, their categorization and definitions were based on time, events in history and physical characteristics as informed by expert judgement, literature and field observations. We achieved this through consultations with village leaders, farmers, pastoralists, land-owners and conservationists regarding the happenings and changes in the area since 1950s or before. Reference is made to around 1950s because the region suffered a catastrophic drought – an aftermath of prolonged rainfall failure following the usual seasonal bushfires that occur in savannas and rangelands^88^. Coupled with stocking and grazing by pastoralists, large parts of the study area have become severely degraded^59,83^.

The five different land cover types were classified as follows: i) pristine grasslands with > 80% grass cover and a history of predominant grass cover for over 70 years; ii) degraded grasslands, i.e. areas with predominantly barren ground for over 70 years; iii) restored grasslands, some 25–35 years old; iv) *Prosopis*-invaded areas with < 30% *Prosopis* cover; and v) *Prosopis*-invaded areas with > 80% *Prosopis* cover. *Prosopis*-low and *Prosopis*-high were invaded some 10–15 years and 25–35 years ago, respectively. Pristine grasslands were characterized by a long history of grass cover, moderate grazing intensity and comprised a mixture of indigenous grasses and shrubs. They were moderately grazed because historically, the pastoralists reserved them as dry season grazing areas and hence carefully regulated grazing during critical seasons^85^. Restored grasslands were barren land which was originally re-seeded with a variety of native grass species, namely; *Cenchrus ciliaris* (L).^89^, *Enteropogon macrostachyus* (Hochst. ex A. Rich.) Munro ex Benth.^90^, *Eragrostis superba (*Peyr).^91^, *Cymbopogon pospischilii* (K. Schum.) C.E. Hubb.^92^ and *Sehima nervosum* (Rottler) Stapf^93^, and experienced moderate grazing pressure, coupled with seasonal harvesting of grass seeds and above-ground biomass. Most of the grass species used are among those recommended by Bogdan and Pratt^94^ for reseeding degraded lands in Kenya. All restored grasslands sites were protected from unauthorised grazing by solar-powered electric fencing. Degraded grasslands were areas with largely barren soil and the plots were selected from communal grazing areas which markedly suffered from severe droughts in the periods before and during the 1950s, 1965^95–97^ and later; and have been unsustainably grazed over time. *Prosopis*-invaded areas were previously degraded grassland and the plots for *Prosopis*-high were selected in areas with the longest history of invasion in the study area, i.e. since the 1980s or early 1990s^31^. We sampled areas with a cover of below 30% and above 80% for plots with a low and high level of *Prosopis* invasion, respectively. These *Prosopis* cover thresholds were determined using a *Prosopis* fractional cover map for the study area (Mbaabu et al., unpublished results).

### Field sampling and laboratory analysis

A preliminary assignment of sites within the study region to the five land cover types was made based on a recently published land use/land cover map. The final decision whether a pre-selected site was sampled or not was taken after inspection of the site in the field and consultation with local people. The selected sites of all land cover types were located in the same geographic region with similar topography, elevation and edaphic characteristics. The patchy distribution of the land cover types in Baringo enabled us to select replicates of the categories that were spatially well interspersed (Fig. 3).

At each selected site, one plot of 15 × 15 m was randomly selected and geo-referenced. Within the pristine and the restored grassland sites, plots were randomly established in areas with contiguous, undisturbed grass cover (i.e. driveways or gullies were excluded) and with a minimum distance of 30 m away from a native tree and 50 m from a *Prosopis* tree or thicket if present. In total, soil samples, plant species richness and herbaceous biomass were analysed in 63 plots (pristine grassland: 10, degraded grassland: 16, *Prosopis*-low: 12, *Prosopis*-high: 10, restored grassland:15; Fig. 3). Soil sampling was carried out during the dry season (September–November 2017 & 2018). Sampling during the non-growing season minimizes the influence of plant type and growth stage on SOC, particularly in soil carbon fractions that turn over rapidly^98^. Plant diversity and herbaceous biomass sampling occurred during the second half of a wet season at plant peak biomass (April-July 2017 & 2018).

The plot design and sampling procedure for all the variables is described in Linders et al.^64^. In brief, each 15 × 15 m plot was divided into nine 5 × 5 m subplots and samples taken from five out of the nine subplots (the four corner subplots and the centre subplot). One of these five subplots was randomly excluded from sampling herbaceous biomass and fenced against livestock grazing in degraded and *Prosopis* invaded plots. We did not find significant differences in herbaceous biomass between fenced and unfenced subplots (TEW Linders, unpublished data). To measure soil organic carbon (SOC), a soil pit was dug incrementally in the centre of each of the corner subplots, from which 4 independent soil cores were taken at the following depth increments: 0–15 cm, 15–30 cm, 30–60 cm and 60–100 cm^99^. For bulk density, a soil pit was dug incrementally in the centre subplot, outside of the fenced area if applicable, and three soil cores taken from three different sites of the same soil pit at each of the same depth increment. Species richness was assessed at the plot level. Herbaceous vegetation (anything growing 2 cm above-ground) samples were harvested from a patch area of 25 × 50 cm of each of the four subplots, pooled together per plot, and oven-dried to determine dry weight^64^. Herbaceous biomass samples were analysed at the Kenya Forestry Research Institute (KEFRI-Nairobi) and the soil samples at the Kenya Agricultural and Livestock Research Organization (KALRO-Kenya). SOC was determined using the colorimetric method^100^. Bulk density samples were oven-dried, weighed and measured using the procedure described by Klute^101^. Bulk density was used to convert the SOC concentration to ecosystem estimates of organic carbon stocks per unit area or soil volume.

### Statistical analysis

All data were analyzed using R^102^, version 3.6.3. We checked for normality and homogeneity using Shapiro–Wilk`s tests and by visual inspections of residuals against fitted values and histograms. Data that violated basic model assumptions were log-transformed prior to the analysis. To assess the effect of soil depth on percent SOC and on SOC per volume soil (g cm^-3^) across land cover types, we fitted linear mixed effect model using the *lme* function within the *nlme* package^103^. We included land cover, soil depth and the interaction of land cover and soil depth as fixed effects in the model, and plot as random factor. Differences between the means among land cover types and soil depths were evaluated with Tukey`s HSD Post-hoc test on the model`s least square means. To assess land cover effects on SOC down to 1 m depth, species richness and herbaceous biomass, we applied general linear models with land cover type as fixed effect, followed by Tukey`s HSD Post-hoc test. Estimates of SOC stocks per unit area (t C ha^-1^) were computed using the formula: SOC t ha^-1^ = %SOC x BD (g cm^-3^) x d (cm), where %SOC = carbon concentration of the sample, BD = bulk density in g cm^-3^, and d = height of the depth increment (cm)^98,104^. Total carbon stocks down to 1 m depth were then obtained by summing up the SOC tons per hectare values estimated for each depth increment^24,105^. To determine which species are characteristic for the different land cover types, we conducted an indicator species analysis test using the *labdsv* package.

When presenting and discussing the results, we adopted a hypothetical, but in the case of Baringo County realistic scenario that pristine grasslands became first degraded and then either were invaded by *Prosopis* or restored. It should be noted that *Prosopis* can also invade pristine grasslands^61^, but historically the majority of the pristine grasslands in Baringo were already degraded at the time *Prosopis* started to spread.

## Supplementary information

Supplementary Information.

## Data Availability

The datasets generated and/or analysed during the current study are available from the corresponding author on reasonable request.
