# Supplementary material for: Restoration of degraded grasslands, but not invasion by Prosopis juliflora, avoids trade-offs between climate change mitigation and other ecosystem services
Source: Sci Rep. 2020 Nov 24;10:20391. doi: 10.1038/s41598-020-77126-7 (PMC7686326; doi:10.1038/s41598-020-77126-7)
Supplement: Supplementary file 1 — Supplementary Information. [file 41598_2020_77126_MOESM1_ESM.docx]

**Supplementary Information**

**Manuscript title:** *Restoration of degraded grasslands, but not invasion by Prosopis juliflora, avoids trade-offs between climate change mitigation and other ecosystem services*

**Authors:** Purity Rima Mbaabu^1, 2, 3*^, Daniel Olago^2^, Maina Gichaba^2^, Sandra Eckert^4^, René Eschen^5^_,_ Silas Oriaso^2^, Simon Kevin Choge^1^, Theo Edmund Werner Linders^5,6,7^, Urs Schaffner^5^

Supplementary Figure S1. SOC per volume (g cm^-3^) for the five land cover types and four soil depth increments. Error bars indicate standard errors. The arrows represent a hypothetical transition from one land cover state to the next over time.

**
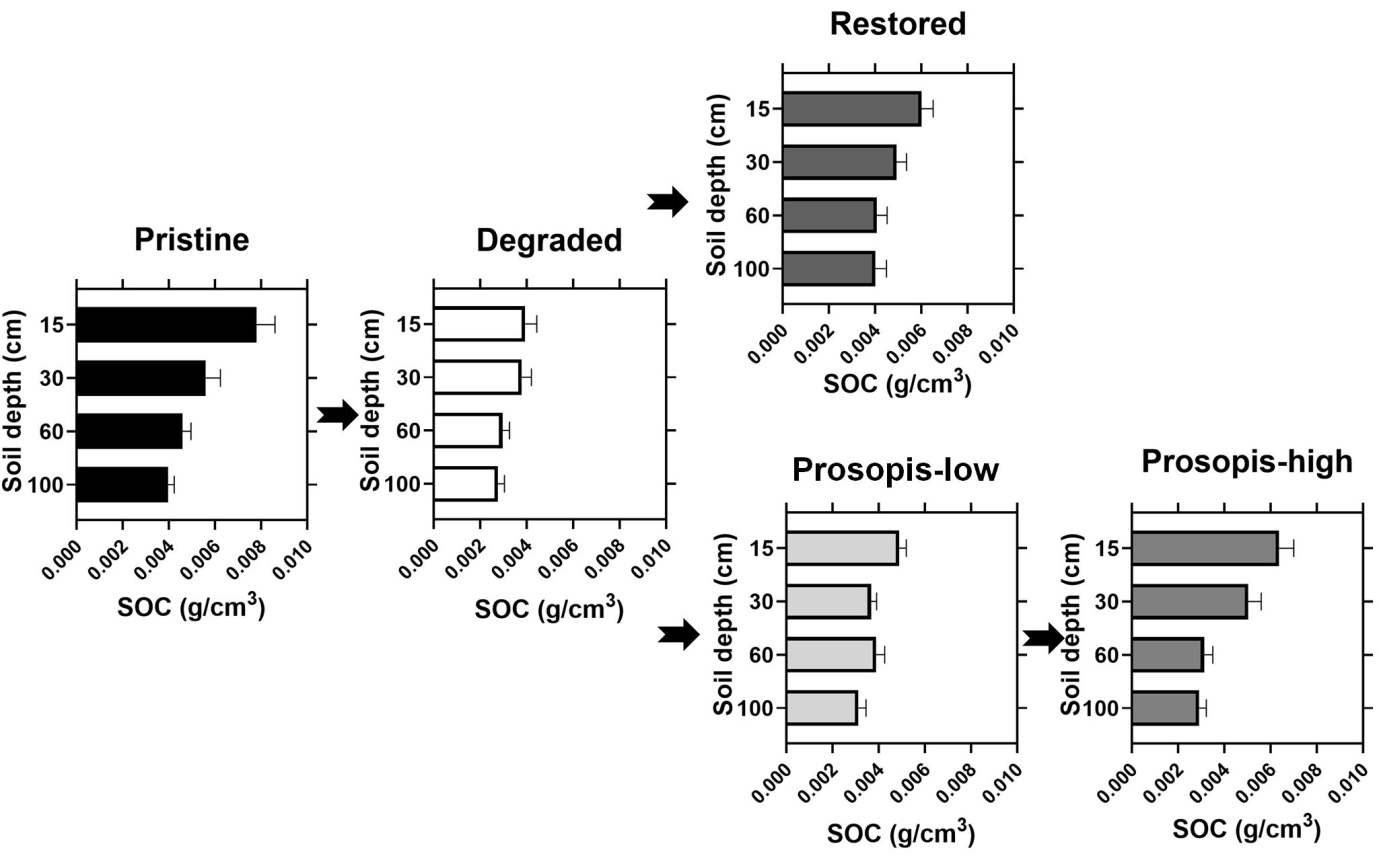
**

Supplementary Figure S2. Interaction effect of land cover type and soil depth on SOC concentration (%SOC).


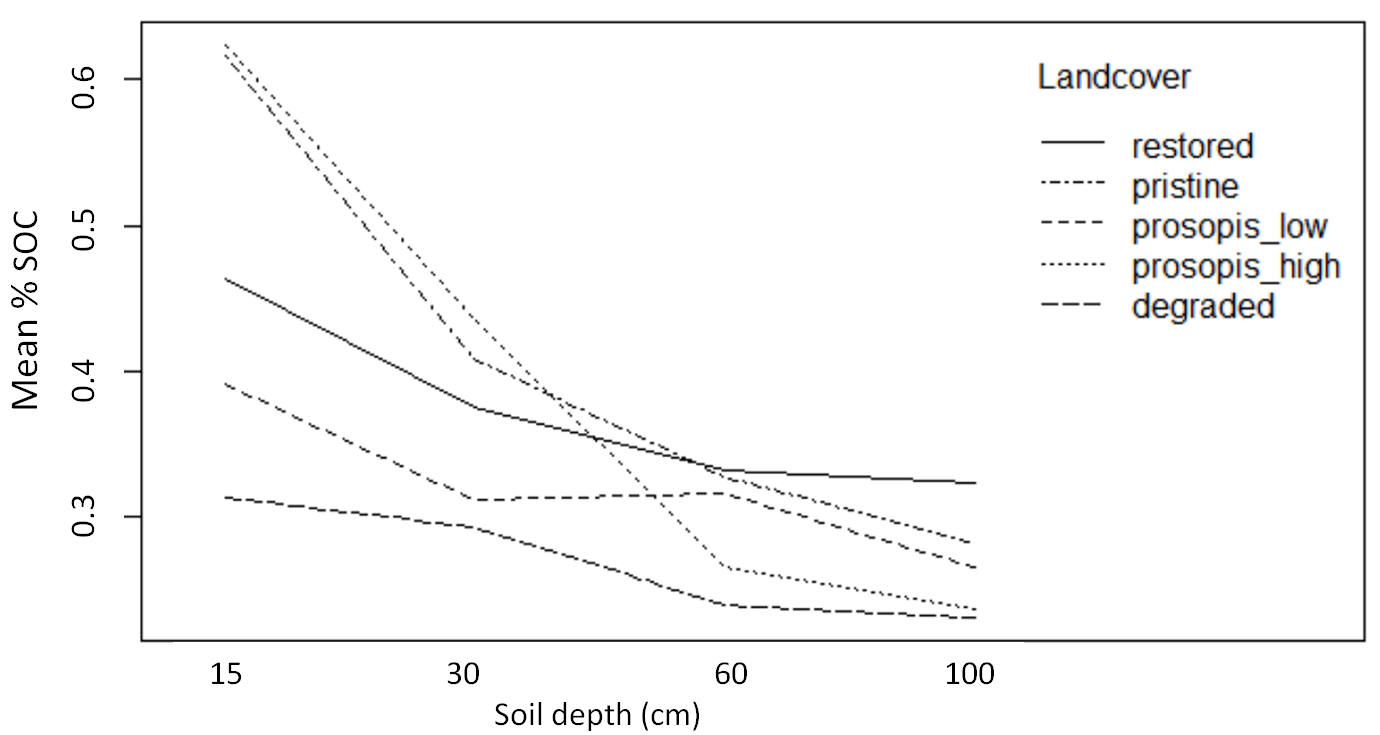


Supplementary Figure S3. Interaction effect of land cover type and soil depth on SOC per volume (SOC g cm^-3^).


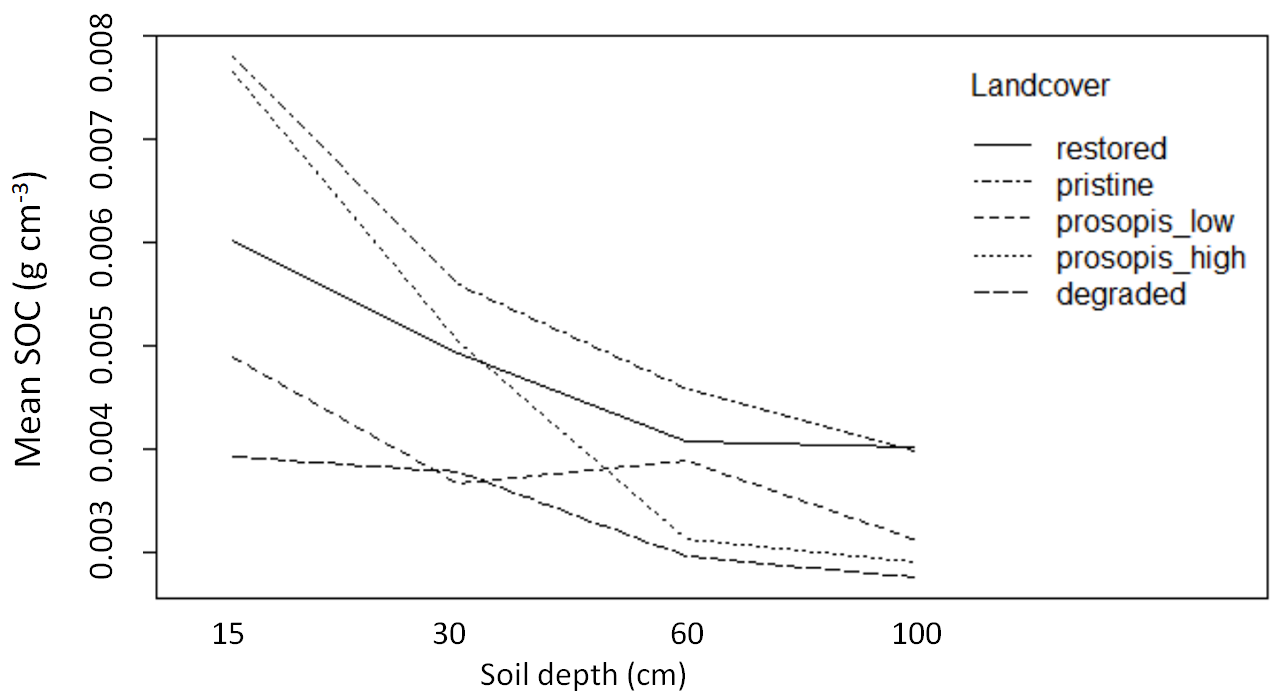


Supplementary Table S1. Effect of land cover type and soil depth on SOC concentration (%SOC). Land cover-soil depth combinations sharing the same letter do not differ significantly from each other (Tukey HSD Post-Doc test; α = 0.05).

|  | | Num DF | | Den DF | | F-value | | | *p-value* |  | |  | | |
| --- | --- | --- | --- | --- | --- | --- | --- | --- | --- | --- | --- | --- | --- | --- |
| (Intercept) | | 1 | | 174 | | 1097.7480 | | | <.0001 |  | |  | | |
| Land cover | | 4 | | 58 | | 4.8207 | | | 0.002 |  | |  | | |
| Soil depth | | 3 | | 174 | | 36.6325 | | | <.0001 |  | |  | | |
| Landcover:soil depth | | 12 | | 174 | | 2.2609 | | | 0.011 |  | |  | | |
|  | |  | |  | |  | | |  |  | |  | | |
| Land cover | Soil depth (cm) | | %SOC | | SE | | df | lower CL | | | upper CL | | Tukey  HSD |  |
| Pristine | 0-15 | | 0.5767 | | 0.0693 | | 58 | 0.3953 | | | 0.8414 | | f |  |
| Degraded | 0-15 | | 0.2816 | | 0.0267 | | 62 | 0.2089 | | | 0.3796 | | abcd |  |
| *Prosopis*-low | 0-15 | | 0.3772 | | 0.0413 | | 58 | 0.2671 | | | 0.5324 | | bcdef |  |
| *Prosopis*-high | 0-15 | | 0.5648 | | 0.0678 | | 58 | 0.3871 | | | 0.8241 | | ef |  |
| Restored | 0-15 | | 0.4483 | | 0.0439 | | 58 | 0.3292 | | | 0.6102 | | def |  |
|  |  | |  | |  | |  |  | | |  | |  |  |
| Pristine | 15-30 | | 0.3870 | | 0.0465 | | 58 | 0.2652 | | | 0.5646 | | bcdef |  |
| Degraded | 15-30 | | 0.2695 | | 0.0256 | | 62 | 0.1999 | | | 0.3633 | | abc |  |
| *Prosopis*-low | 15-30 | | 0.3044 | | 0.0333 | | 58 | 0.2156 | | | 0.4297 | | abcd |  |
| *Prosopis*-high | 15-30 | | 0.3967 | | 0.0476 | | 58 | 0.2718 | | | 0.5787 | | cdef |  |
| Restored | 15-30 | | 0.3539 | | 0.0347 | | 58 | 0.2599 | | | 0.4817 | | bcdef |  |
|  |  | |  | |  | |  |  | | |  | |  |  |
| Pristine | 30-60 | | 0.3188 | | 0.0383 | | 58 | 0.2185 | | | 0.4652 | | abcde |  |
| Degraded | 30-60 | | 0.2234 | | 0.0212 | | 62 | 0.1657 | | | 0.3012 | | ab |  |
| *Prosopis*-low | 30-60 | | 0.3019 | | 0.0331 | | 58 | 0.2138 | | | 0.4262 | | abcd |  |
| *Prosopis*-high | 30-60 | | 0.2456 | | 0.0295 | | 58 | 0.1683 | | | 0.3583 | | ab |  |
| Restored | 30-60 | | 0.3047 | | 0.0298 | | 58 | 0.2238 | | | 0.4148 | | abcd |  |
|  |  | |  | |  | |  |  | | |  | |  |  |
| Pristine | 60-100 | | 0.2786 | | 0.0334 | | 58 | 0.1909 | | | 0.4064 | | abcd |  |
| Degraded | 60-100 | | 0.2101 | | 0.0199 | | 62 | 0.1558 | | | 0.2832 | | a |  |
| *Prosopis*-low | 60-100 | | 0.2504 | | 0.0274 | | 58 | 0.1773 | | | 0.3535 | | abc |  |
| *Prosopis*-high | 60-100 | | 0.2273 | | 0.0273 | | 58 | 0.1558 | | | 0.3317 | | ab |  |
| Restored | 60-100 | | 0.2942 | | 0.0288 | | 58 | 0.2161 | | | 0.4005 | | abc |  |

Supplementary Table S2. Effect of land cover type and soil depth on SOC per volume (SOC g cm^-3^). Land cover-soil depth combinations sharing the same letter do not differ significantly from each other (Tukey HSD Post-Doc test; α = 0.05).

|  | | Num DF | | | Den DF | | | F-value | | | | *p-value* | | |  |  | | |
| --- | --- | --- | --- | --- | --- | --- | --- | --- | --- | --- | --- | --- | --- | --- | --- | --- | --- | --- |
| (Intercept) | | 1 | | | 174 | | | 25021.776 | | | | <.0001 | | |  |  | | |
| Land cover | | 4 | | | 58 | | | 6.148 | | | | 0.0003 | | |  |  | | |
| Soil depth | | 3 | | | 174 | | | 37.796 | | | | <.0001 | | |  |  | | |
| Land cover:soil depth | | 12 | | | 174 | | | 1.981 | | | | 0.0285 | | |  |  | | |
|  |  | |  | | | |  | | |  | | |  | | |  | |  |
| Land cover | Soil depth (cm) | | | SOC per  volume | | SE | | | df | | lower CL | | | upper CL | | | Tukey  HSD | |
| Pristine | 0-15 | | | 0.0074 | | 0.0009 | | | 58 | | 0.0051 | | | 0.0109 | | | k | |
| Degraded | 0-15 | | | 0.0035 | | 0.0003 | | | 62 | | 0.0026 | | | 0.0048 | | | abcdefghi | |
| *Prosopis*-low | 0-15 | | | 0.0048 | | 0.0005 | | | 58 | | 0.0034 | | | 0.0068 | | | cdfhijk | |
| *Prosopis*-high | 0-15 | | | 0.0069 | | 0.0008 | | | 58 | | 0.0047 | | | 0.0101 | | | jk | |
| Restored | 0-15 | | | 0.0057 | | 0.0005 | | | 58 | | 0.0042 | | | 0.0078 | | | ijk | |
|  |  | | |  | |  | | |  | |  | | |  | | |  | |
| Pristine | 15-30 | | | 0.0053 | | 0.0006 | | | 58 | | 0.0036 | | | 0.0077 | | | ghijk | |
| Degraded | 15-30 | | | 0.0035 | | 0.0003 | | | 62 | | 0.0026 | | | 0.0048 | | | abcdefghi | |
| *Prosopis*-low | 15-30 | | | 0.0036 | | 0.0003 | | | 58 | | 0.0025 | | | 0.0051 | | | abcdefghi | |
| *Prosopis*-high | 15-30 | | | 0.0047 | | 0.0005 | | | 58 | | 0.0032 | | | 0.0068 | | | efghijk | |
| Restored | 15-30 | | | 0.0046 | | 0.0005 | | | 58 | | 0.0034 | | | 0.0063 | | | bdghijk | |
|  |  | | |  | |  | | |  | |  | | |  | | |  | |
| Pristine | 30-60 | | | 0.0044 | | 0.0005 | | | 58 | | 0.0030 | | | 0.0065 | | | abcdefghij | |
| Degraded | 30-60 | | | 0.0028 | | 0.0003 | | | 62 | | 0.0021 | | | 0.0037 | | | acef | |
| *Prosopis*-low | 30-60 | | | 0.0036 | | 0.0004 | | | 58 | | 0.0026 | | | 0.0052 | | | abcdefghi | |
| *Prosopis*-high | 30-60 | | | 0.0029 | | 0.0004 | | | 58 | | 0.0020 | | | 0.0043 | | | abcdefgh | |
| Restored | 30-60 | | | 0.0037 | | 0.0004 | | | 58 | | 0.0027 | | | 0.0050 | | | abcdefgh | |
|  |  | | |  | |  | | |  | |  | | |  | | |  | |
| Pristine | 60-100 | | | 0.0039 | | 0.0005 | | | 58 | | 0.0027 | | | 0.0057 | | | abcdefghij | |
| Degraded | 60-100 | | | 0.0026 | | 0.0002 | | | 62 | | 0.0019 | | | 0.0035 | | | a | |
| *Prosopis*-low | 60-100 | | | 0.0029 | | 0.0003 | | | 58 | | 0.0021 | | | 0.0041 | | | abeg | |
| *Prosopis*-high | 60-100 | | | 0.0028 | | 0.0003 | | | 58 | | 0.0019 | | | 0.0040 | | | abcd | |
| Restored | 60-100 | | | 0.0036 | | 0.0004 | | | 58 | | 0.0027 | | | 0.0049 | | | abcdefgh | |

Supplementary Table S3. Effect of land cover type and soil depth on bulk density (g cm^-3^). Land cover-soil depth combinations sharing the same letter do not differ significantly from each other (Tukey HSD Post-Doc test; α = 0.05).

|  | | Num DF | | | Den DF | | | F-value | | | | *p-value* | | |  |  | | |
| --- | --- | --- | --- | --- | --- | --- | --- | --- | --- | --- | --- | --- | --- | --- | --- | --- | --- | --- |
| (Intercept) | | 1 | | | 174 | | | 11326.942 | | | | <.0001 | | |  |  | | |
| Land cover | | 4 | | | 58 | | | 5.106 | | | | 0.0014 | | |  |  | | |
| Soil depth | | 3 | | | 174 | | | 1.549 | | | | 0.2036 | | |  |  | | |
| Land cover:soil depth | | 12 | | | 174 | | | 2.482 | | | | 0.0051 | | |  |  | | |
|  |  | |  | | | |  | | |  | | |  | | |  | |  |
| Land cover | Soil depth (cm) | | | Bulk  density | | SE | | | df | | lower CL | | | upper CL | | | Tukey  HSD | |
| Pristine | 0-15 | | | 1.30 | | 0.0392 | | | 58 | | 1.18 | | | 1.42 | | | abc | |
| Degraded | 0-15 | | | 1.26 | | 0.0310 | | | 62 | | 1.17 | | | 1.36 | | | abc | |
| *Prosopis*-low | 0-15 | | | 1.27 | | 0.0358 | | | 58 | | 1.16 | | | 1.38 | | | abc | |
| *Prosopis*-high | 0-15 | | | 1.22 | | 0.0392 | | | 58 | | 1.10 | | | 1.35 | | | abc | |
| Restored | 0-15 | | | 1.29 | | 0.0320 | | | 58 | | 1.19 | | | 1.39 | | | abc | |
|  |  | | |  | |  | | |  | |  | | |  | | |  | |
| Pristine | 15-30 | | | 1.37 | | 0.0392 | | | 58 | | 1.25 | | | 1.49 | | | bc | |
| Degraded | 15-30 | | | 1.30 | | 0.0310 | | | 62 | | 1.20 | | | 1.40 | | | abc | |
| *Prosopis*-low | 15-30 | | | 1.18 | | 0.0358 | | | 58 | | 1.07 | | | 1.29 | | | ab | |
| *Prosopis*-high | 15-30 | | | 1.18 | | 0.0392 | | | 58 | | 1.06 | | | 1.31 | | | ab | |
| Restored | 15-30 | | | 1.31 | | 0.0320 | | | 58 | | 1.21 | | | 1.41 | | | abc | |
|  |  | | |  | |  | | |  | |  | | |  | | |  | |
| Pristine | 30-60 | | | 1.40 | | 0.0392 | | | 58 | | 1.27 | | | 1.52 | | | c | |
| Degraded | 30-60 | | | 1.25 | | 0.0310 | | | 62 | | 1.15 | | | 1.35 | | | abc | |
| *Prosopis*-low | 30-60 | | | 1.23 | | 0.0358 | | | 58 | | 1.12 | | | 1.34 | | | abc | |
| *Prosopis*-high | 30-60 | | | 1.16 | | 0.0392 | | | 58 | | 1.04 | | | 1.29 | | | a | |
| Restored | 30-60 | | | 1.22 | | 0.0320 | | | 58 | | 1.12 | | | 1.32 | | | abc | |
|  |  | | |  | |  | | |  | |  | | |  | | |  | |
| Pristine | 60-100 | | | 1.40 | | 0.0392 | | | 58 | | 1.28 | | | 1.52 | | | c | |
| Degraded | 60-100 | | | 1.23 | | 0.0310 | | | 62 | | 1.13 | | | 1.32 | | | abc | |
| *Prosopis*-low | 60-100 | | | 1.17 | | 0.0358 | | | 58 | | 1.06 | | | 1.28 | | | a | |
| *Prosopis*-high | 60-100 | | | 1.22 | | 0.0392 | | | 58 | | 1.10 | | | 1.35 | | | abc | |
| Restored | 60-100 | | | 1.23 | | 0.0320 | | | 58 | | 1.13 | | | 1.33 | | | abc | |

Supplementary Table S4. Effect of land cover type on total SOC per unit area (t ha^-1^). Land cover types sharing a letter are not significantly different from each other (Tukey HSD Post-Doc test; α = 0.05).

|  | Df | Sum of Squares | | Mean Square | | F-value | p-value | |
| --- | --- | --- | --- | --- | --- | --- | --- | --- |
| Landcover | 4 | 2539 | | 634.7 | | 5.532 | 0.000766*** | |
| Residuals | 58 | 6654 | | 114.7 | |  |  | |
|  |  |  | |  | |  |  | |
| Land cover | total SOC | SE | Asymp.LCL | | Asymp.UCL | | Tukey HSD |  |
| Degraded | 31.52287 | 2.677832 | 24.64416 | | 38.40159 | | a |  |
| *Prosopis*-low | 36.98844 | 3.092094 | 29.04558 | | 44.93130 | | ab |  |
| *Prosopis*-high | 40.04675 | 3.387220 | 31.34578 | | 48.74772 | | abc |  |
| Restored | 44.67903 | 2.765653 | 37.57473 | | 51.78334 | | bc |  |
| Pristine | 49.75775 | 3.387220 | 41.05678 | | 58.45872 | | c |  |

Supplementary Table S5. Effect of land cover type on plant species richness (number of species plot^-1^). Land cover types sharing a letter do not differ significantly from each other (Tukey HSD Post-Doc test; α = 0.05).

|  | Df | | Sum of Squares | Mean Square | F-value | p-value |
| --- | --- | --- | --- | --- | --- | --- |
| Land cover | 4 | | 6.245 | 1.5612 | 8.656 | <0.0001*** |
| Residuals | 58 | | 10.461 | 0.1804 |  |  |
|  | | | | | | |
| Land cover | | species richness | SE | Asymp.LCL | Asymp.UCL | Tukey HSD |
| Degraded | | 7.23 | 0.77 | 5.50 | 9.49 | a |
| Restored | | 8.59 | 0.94 | 6.48 | 11.38 | ab |
| *Prosopis*-high | | 8.97 | 1.20 | 6.35 | 12.66 | ab |
| *Prosopis*-low | | 12.26 | 1.50 | 8.94 | 16.79 | bc |
| Pristine | | 18.21 | 2.45 | 12.90 | 25.72 | c |
|  |  | |  |  |  |  |

Supplementary Table S6. Effect of land cover type on dry herbaceous biomass (dry weight g m^-2^). Land cover types sharing a letter do not differ significantly from each other (Tukey HSD Post-Doc test; α = 0.05).

|  | Df | | Sum of Squares | Mean Square | F-value | p-value |
| --- | --- | --- | --- | --- | --- | --- |
| Land cover | 4 | | 14578 | 3644 | 33.97 | <0.0001*** |
| Residuals | 58 | | 6223 | 107 |  |  |
|  | | | | | | |
| Land cover | | Biomass | SE | Asymp.LCL | Asymp.UCL | Tukey HSD |
| *Prosopis*-high | | 13.90 | 3.28 | 5.49 | 22.31 | a |
| *Prosopis*-low | | 21.29 | 2.99 | 13.61 | 28.97 | a |
| Degraded | | 23.03 | 2.59 | 16.38 | 29.68 | a |
| Restored | | 46.73 | 2.67 | 39.86 | 53.60 | b |
| Pristine | | 55.20 | 3.28 | 46.79 | 63.61 | b |

Supplementary Table S7. Aboveground carbon stock (mean and standard deviation) expressed in tonnes per hectare for pristine, degraded, restored and Prosopis invaded areas. These were determined using dry woody Prosopis aboveground biomass (for Prosopis invaded areas) derived by applying a Prosopis-species allometric equation on the diameter (at 0.3 m from ground) of all individual Prosopis trees in randomly sampled 5x5 m subplots following the methods described by Linders et al.,^1^. The Prosopis species allometric equation was developed by Linders et al.,^1^ using Prosopis data from Kenya by Muturi et al.,^2^. For grasslands and degraded areas, dry herbaceous biomass was used to estimate aboveground carbon stocks. For all land cover types, a conversion factor of 0.47 was used to convert dry aboveground biomass to carbon stocks^3^. For Prosopis, aboveground carbon was estimated from a subset of Prosopis-low and Prosopis-high plots where we have aboveground woody biomass data.

| Land cover | n | Above-ground C (t ha^-1^) | SD |
| --- | --- | --- | --- |
| Pristine | 10 | 6.02 | 3.17 |
| Degraded | 16 | 0.69 | 0.81 |
| Restored | 15 | 3.19 | 1.55 |
| Prosopis-high | 5 | 12.46 | 6.36 |
| Prosopis-low | 4 | 3.00 | 1.68 |

References

1.  Linders, T. E. W. et al. The Impact of Invasive Species on Social-Ecological Systems: Relating Supply and Use of Selected Provisioning Ecosystem Services. *Ecosyst. Serv.* **41**, 101055; https://doi.org/10.1016/j.ecoser.2019.101055 (2020).

2.  Muturi, G. M., Kariuki, J. G., Poorter, L. & Mohren, G. M. J. Allometric Equations for Estimating Biomass in Naturally Established Prosopis Stands in Kenya. *J. Hortic. For.* **4**, (4); https://doi.org/10.5897/JHF11.066 (2012).

3.  IPCC. Chapter 2. Generic Methodologies Applicable to Multiple Land-Use Categories. In *In: 2006 IPCC Guidelines for National Greenhouse Gas Inventories.*; Eggleston, H., Buendia, L., Miwa, K., Ngara, T., Tanabe, K., Eds.; Agriculture, forestry and other land use.; IGES, Japan, **4**, p 2.1-2.59 (2006).
